# Topical Application of Retinyl Palmitate-Loaded Nanotechnology-Based Drug Delivery Systems for the Treatment of Skin Aging

**DOI:** 10.1155/2014/632570

**Published:** 2014-03-19

**Authors:** Marcela B. Oliveira, Alice Haddad do Prado, Jéssica Bernegossi, Claudia S. Sato, Iguatemy Lourenço Brunetti, Maria Virgínia Scarpa, Gislaine Ricci Leonardi, Stig E. Friberg, Marlus Chorilli

**Affiliations:** ^1^Departament of Drugs and Pharmaceuticals, School of Pharmaceutical Sciences, UNESP, 14801-902 Araraquara, SP, Brazil; ^2^Departament of Clinical Analysis, School of Pharmaceutical Sciences, UNESP, 14801-902 Araraquara, SP, Brazil, Brazil; ^3^Institute of Environmental, Chemistry and Pharmaceuticals Sciences, Federal University of São Paulo (UNIFESP), 09972-270 Diadema, SP, Brazil; ^4^Ugelstad Laboratory, Norwegian University of Science and Technology (NTNU), 7491 Trondheim, Norway

## Abstract

The objective of this study was to perform a structural characterization and evaluate the* in vitro* safety profile and* in vitro* antioxidant activity of liquid crystalline systems (LCS) with and without retinyl palmitate (RP). LCS containing polyether functional siloxane (PFS) as a surfactant, silicon glycol copolymer (SGC) as oil phase, and water in the ratios 30 : 25 : 45 and 40 : 50 : 10 with (OLS_*v*_ = RP-loaded opaque liquid system and TLS_*v*_ = RP-loaded transparent liquid system, respectively) and without (OLS and TLS, respectively) RP were studied. Samples were characterized using polarized light microscopy (PLM) and rheology analysis. *In vitro* safety profile was evaluated using red cell hemolysis and *in vitro* cytotoxicity assays. *In vitro* antioxidant activity was performed by the DPPH method. PLM analysis showed the presence of lamellar LCS just to TLS. Regardless of the presence of RP, the rheological studies showed the pseudoplastic behavior of the formulations. The results showed that the incorporation of RP in LCS improved the safety profile of the drug.* In vitro* antioxidant activity suggests that LCS presented a higher capacity to maintain the antioxidant activity of RP. PFS-based systems may be a promising platform for RP topical application for the treatment of skin aging.

## 1. Introduction

Currently, the demand for products that reduce skin aging is constantly growing because people want to stay young as long as possible. Cosmeceuticals (cosmetics producing beneficial results for the body, for example, the effects of antifree radicals) represent one of the largest growing segments of the skin care market, especially for products that are designed to prevent and treat skin aging [1, 2].

Aging is a natural and inevitable process that reverses the biological characteristics acquired during development and leads to cell death [3]. The natural process of skin rejuvenation slows dramatically and the skin becomes thinner and drier, losing elasticity [4]. Skin aging is influenced by several factors including genetic factors, environmental exposures (ultraviolet radiation (UV), xenobiotics, and stress mechanisms), hormonal changes, and metabolic processes (generation of reactive oxygen species as chemically activated sugars and aldehydes). All of these factors act together in changing the structure, function, and appearance of the skin [5].

The use of retinoids has been highlighted for the treatment of photoaging, and there are many different brands and formulations currently available in the market that employ such substances. A randomized clinical study showed beneficial results of retinoids in photoaging, in addition to repairing and preventing skin aging [6].

Studies have indicated that retinoids may have specific effects on the receptor resulting in decreased skin roughness and skin facial wrinkles [7]. A study with 24 Korean women for 24 weeks showed improvement in skin roughness and fine wrinkles [8, 9].

**Figure 1:**
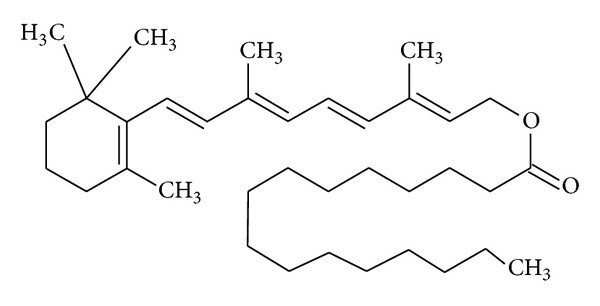
Molecular structure of retinyl palmitate.

Retinyl palmitate, whose molecular structure is shown in Figure 1, is an ester of retinol and is the major form of vitamin A found in the epidermis. This compound has been widely used in pharmaceutical and cosmetic formulations [3]. It has a high molecular weight and a stable formulation. To be active, RP should be enzymatically converted in the skin to retinol by cleavage of the ester linkage and must then be converted to tretinoin via oxidative processes. The topical administration of RP for 14 days in rats resulted in increased protein and collagen and an epidermal thickening [6].

The main obstacle to the use of topical retinoids is the high incidence of skin irritation. Patients may develop dermatitis with redness and tenderness of the skin. This usually occurs within two to four weeks after initiation of treatment and usually disappears when the treatment is continued. Nevertheless, many patients discontinue therapy because of these reactions. It has been found that derivatives of retinol such as RP do not produce the same irritant effects as retinoic acid and induce the same cellular and molecular changes observed with the application of retinoic acid [10].

Sorg et al. (2005) demonstrated that, although the amounts of RP naturally present in the epidermis are too low to provide effective and efficient protection against UV radiation, these retinoids can easily be administered topically in order to promote effective protection. This was confirmed by some studies, including one conducted in human volunteers who were subjected to UVB light to assess DNA damage and erythema. The participants were treated with a commercial sunscreen (octyl methoxycinnamate) and one containing RP, and it was observed that RP inhibited the formation of thymine dimers (an indicator of DNA damage) and erythema [3]. Another study showed that DNA damage and apoptosis were inhibited in mice by the application of topical retinoic acid, retinaldehyde, retinol, and RP [11].

Currently, the development of pharmaceutical and cosmetic technology is not restricted only to the discovery of new molecules but also to the development of new systems to deliver active ingredients or optimize their release [12]. The combination of consumer desire and the development of these technologies has led to the emergence of new systems with specific properties, capable of incorporating substances with different profile releases optimized for application on different areas [13]. Among the systems developed or with applicability for topical skincare cosmetics, nanotechnology-based drug delivery systems are promising vehicles for dermal and transdermal release of active compounds, particularly the liquid crystalline systems (LCS), multiple emulsions, and nanoemulsions [13, 14]. LCS have applications in cosmetic and pharmaceutical formulations. They are able to promote the encapsulation of active ingredients, allowing for sustained release of the same, as well as giving protection of drugs and photosensitivity depending on the microstructure, increasing the stability of these systems to reduce coalescence and change in viscosity [13, 15, 16].

Furthermore, LCS can maintain therapeutic response for a longer period of time, improve drug efficacy and solubility, decrease side effects, and interfere in skin hydration. The incorporation of RP within LCS phases provided a significant reduction in the orbicular wrinkles of human volunteers [17].

Studies involving nanotechnology seek to implement new technological approaches to extend the benefits provided by drug delivery systems. The development of an effective, safe, and reliable carrier system which exhibits good bioavailability and pharmacodynamics and also reduces side effects is a goal for many researchers. Therefore, finding a system that meets those requirements would be extremely valuable and SLC are potential candidates [13, 18].

The objective of the present study was to perform a structural characterization and evaluate the* in vitro *safety profile and antioxidant activity of lamellar LCS, with and without RP.

## 2. Materials and Methods

### 2.1. Materials

Polyether functional siloxane, DC 5329 (S), and silicon glycol copolymer, DC 193 (O), were purchased from Dow Corning (Michigan, USA), and retinyl palmitate (RP) 1,000,000 IU/g was purchased from Roche (Greenzach-Wyhlen, Germany). High purity water (W) from a Millipore Milli-Q plus purification system was used throughout.

### 2.2. Formulation Preparation

The preparation has been described previously [17]. The samples were prepared by heating a mixture of O and S to 45°C. W was heated to 40°C and then carefully added under gentle and constant stirring until the mixture reached room temperature. The resulting systems, containing various proportions of the components, were characterized using a pseudoternary phase diagram in order to define the proportions that form LCS [17]. The proportions of each component were calculated from titrations of the binary mixtures of oil phase and surfactant with water. The transitions from an opaque semisolid phase to a transparent viscous system (TVS), viscous and opaque system (OVS), and an opaque liquid system (OLS), as well as a transparent liquid system (TLS) and phase separation (PS), were defined. Diagrams were produced for the RP-loaded (1%) and RP-unloaded mixtures.

### 2.3. Polarized Light Microscopy

A small amount of the formulations was placed on a glass slides, covered with a cover slip, and examined with the aid of a polarized light microscope (Jenamed 2, Carl Zeiss-Jena) to judge the homogeneity of the dispersions and the presence of optically anisotropic areas by the patterns formed with the sample between crossed polarizers.

### 2.4. Rheology Analysis

The rheological determination of formulations was carried out with a controlled-stress rheometer (model RS-1, Haake RheoStress) with plate-plate geometry. This geometry consists of two stainless steel plates 2 cm in diameter with a gap of 200 *μ*m between the plates. Samples were carefully applied to the lower plate, ensuring that formulation shearing was minimized, and allowed to equilibrate for at least 3 minutes prior to analysis. The experiments were carried out with shear rates in the range of 0.001–30 s^−1^. The shear rate region used was selected on the basis of the strength of resistance to the applied stresses. The rheological measurements were performed on both the up and down curve. All rheological determinations were carried out on all samples at 25.0 ± 0.2°C.

### 2.5. *In Vitro* Biological Assays

#### 2.5.1. Erythrocyte Hemolysis

The erythrocyte hemolysis assay was performed using the experimental procedure described by Jumaa et al. [19] and Huang et al. [20]. Briefly, before use, freshly collected human blood (O positive) was washed three times with 0.01 M Tris-HCl with a pH 7.4 containing 0.15 M NaCl (Tri-saline). A suspension of 1% (v/v) erythrocytes was prepared with packed red blood cells resuspended in Tris-saline. RP-loaded LCS, RP-unloaded LCS, and RP free were dissolved in Tris-saline to a final concentration of 27 *μ*M. As a positive control (100% lysis), a 1% (v/v) Triton X-100 solution was used. After incubation for 1 hour at 37°C, the samples were centrifuged at 3000 ×g for 2 minutes. Aliquots of 100 *μ*L of the supernatant were transferred to 96-well microplates, and the absorbance was determined at 405 nm using a BioRad Model 3550-UV (USA) microplate reader. The assay was performed in triplicate. The percentage of hemolysis was calculated using the following equation: % hemolysis = (absorbance of the test sample/absorbance at 100% lysis) × 100.

#### 2.5.2. *In Vitro* Nonspecific Cytotoxicity

*In vitro* testing for analysis of the cytotoxicity of the formulations was performed using J-774 mouse macrophages as template. Cells were seeded in bottom microplates (Nunclon) with 96 wells and a density of 2.5–10.0 × 10^5^ cells/well with different doses of the formulation and free RP (18.6, 10, 5, and 1 *μ*M) or vehicle control for 48 hours. The cells were washed with PBS after removal of the compounds and cell viability was assessed by colorimetry formazan (MTT).

The method of 3 [4,5-dimethylthiazol-2-yl]-2,5-diphenyltetrazolium bromide (MTT) is simple, reliable, and reproducible colorimetric method for measuring mitochondrial metabolic reduction of yellow tetrazolium salt to the insoluble formazan crystals in aqueous solution of viable cells. Cells and MTT (0.4 mg/mL) were incubated at 37°C for 3 hours. Subsequently, the supernatant was removed and formazan crystals were dissolved in DMSO (180 mL). The plates were agitated for 10 minutes and optical density was measured using a multiwall spectrophotometer operating at 560 nm. Concentrations were tested in triplicate using six additional controls (cells in medium). Cell viability was calculated using the following equation: cell viability (%) = [OD_560_ (sample)/OD_560_ (control)] × 100.

### 2.6. *In Vitro* Antioxidant Activity

Free radical scavenging activity was evaluated by the 2,2-diphenyl-1-picrylhydrazyl (DPPH) test with modifications [21]. One hundred microliters of RP free, formulations, or control (ethanol; 10–60 *μ*g/mL) was added to 3.9 mL of DPPH solution (ethanol; 60 *μ*M). After 30 minutes storage in a dark place, the absorbance measures were calculated using a spectrophotometer at 517 nm. All measurements were repeated three times. Free radical scavenging activity was calculated using the following formula: % inhibition DPPH = [(*A*_0_ − *A*_1_)/*A*_0_ × 100], where *A*_0_ represents absorbance of control and *A*_1_ represents absorbance of sample. The IC_50_ value was determined by plotting concentration of formulations* versus* the percentage keeping DPPH at a steady state [21].

### 2.7. Statistical Analyses

Data were analyzed using the mean and standard deviation and compared by analysis of variance (ANOVA). The Tukey test was used to assess significant differences between samples, where values *P* < 0.05 were considered statistically significant. The program Origin 7.0 SRO was used for the treatment of the data.

## 3. Results and Discussion

**Figure 2:**
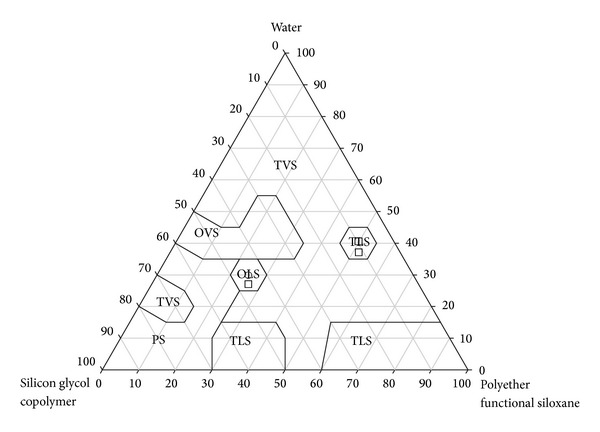
Ternary phase diagram, with ⚪ and the square (□) representing the selected regions, where TVS is transparent viscous system, OVS is opaque and viscous system, TLS is transparent liquid system, OLS is opaque liquid system, and PS is phase separation.

Formulations were prepared with different surfactant/oil/water ratios, with and without RP. The compositions were based on the pseudoternary phase diagram previously constructed for the same mixture in the experimental conditions [16]. Two formulations were selected for tests—OLS, constituted by 30% surfactant (polyether functional siloxane, PSF), 25% of oil phase (silicon glycol copolymer, SGC), and 45% of water, and TLS—constituted by 40% PSF, 50% SGC, and 10% water. To each of these systems, 1% RP was added, yielding the formulations OLS_*v*_ and TLS_*v*_. The development and characterization of the system were crucial to the choice of the formulation study. The ternary phase diagram (Figure 2) obtained had extensive concentration transparent systems (TVS and TLS), since the oil phase of the system also has surfactant properties. At low concentrations of water and oil intermediates OVS and OLS were formed and concentrations of oil and water phase below 30% were observed PS.

**Figure 3:**
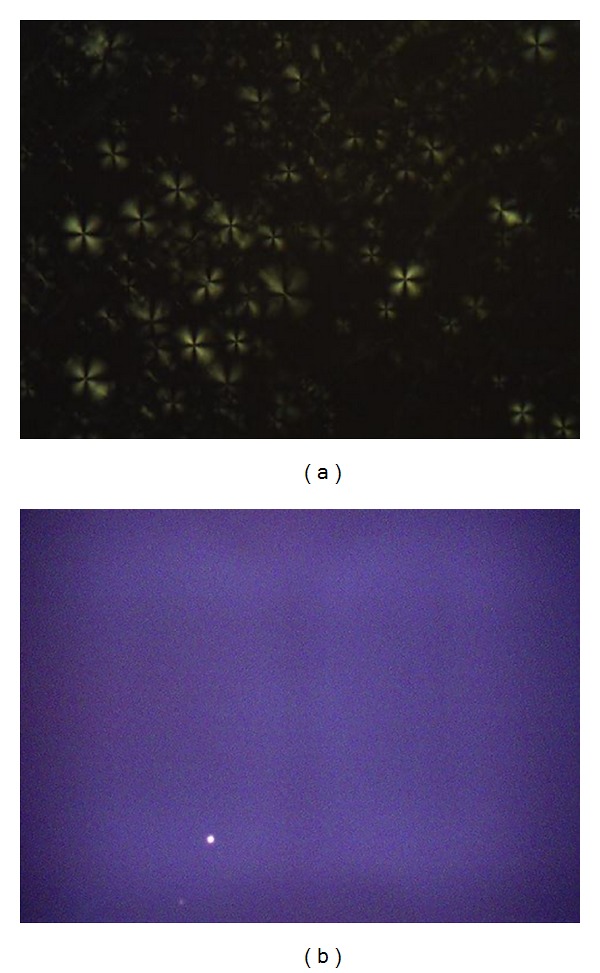
Images obtained by Polarized light microscopy: (a) transparent liquid system (TLS) showed Maltese crosses; (b) opaque liquid system (OLS) showed dark field. The objects are air bubbles (magnified of 20x).

The determination of the optical properties of the formulations was performed observing the sample between crossed polarizers. Lamellar and hexagonal mesophases are anisotropic, while cubic mesophases are isotropic [22, 23]. The presence of Maltese crosses for TLS, Figure 3(a), showed the presence of a lamellar liquid crystal, LCS. The formulation OLS did not present liquid crystalline mesophases, as shown by the homogenous dark field (Figure 3(b)) [18].

**Figure 4:**
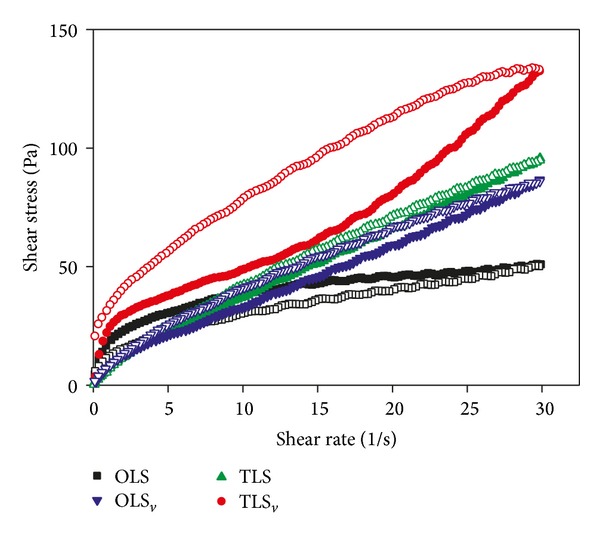
Rheogram of the systems studied, where OLS is opaque liquid system, OLS_*v*_ is RP-loaded opaque liquid system, TLS is transparent liquid system, and TLS_*v*_ is RP-loaded transparent liquid system. The solid symbols represent the upper curves and the hollow symbols represent the lower curves.

In the pharmaceutical development of cosmetics, the study of rheology (flow characteristics) has fundamental importance when considering the manufacturing process and the preparation, transportation, storage, and use by consumers [24, 25]. The rheological analysis was made in the form of rheograms, showing the relationship between shear stress (*σ*) and shear rate (*γ*). In Figure 4 we can observe the relationship between *σ* and *γ* for all of the formulations. For some combinations the values of the shear stress are different for the curves with increased and reduced shear rate. This characteristic is typical of thixotropic materials [26].

The viscosity decreases with increasing shear rate which occurred in the studied system irrespective of the definition of viscosity used and is a characteristic of pseudoplastic fluid [26]. This makes the product suitable for topical use, because of the initial shear to apply the sample; the reduced viscosity results in a good spreading during application and in the formation of uniform film on the skin surface [27–29]. In the formulations, by decreasing shear rate the values return to baseline, the rheological behavior is reversible, and the viscosity returns to the initial value sometime after the initial step deformation [30–33]. This phenomenon is also useful for skin applications, since the increased viscosity assists in retaining the formulation in place. As for the OLS sample compared with sample TLS a pronounced decrease in viscosity was observed with increasing shear rate. For both formulations, the addition of vitamin increased the viscosity of the formulations.

The behavior of these formulations can be reaffirmed based on power law, described in the following equation:  *τ* = *k* · *γ* ^*η*^, where *k* and *η* values are related to the consistency and flow index, respectively. Thus, in this model, values of *n* greater than 1 represent dilatant fluid, values of *n* smaller than 1 represent a pseudoplastic fluid, and finally values of *n* equal to 1 represent Newtonian fluid [34].

In Table 1, the values of *η* and *k* for all formulations as compared to the rating of the rheogram are presented.

The formulations outlined in Table 1 (OLS, OLS_*v*_, TLS, and TLS_*v*_) show that all values of *η* are smaller than 1, indicating pseudoplastic fluids which agree with the interpretation based on the observation of the rheogram of Figure 4. In OLS, after adding RP, there was an increase in the value of *η*, from 0.28834 to 0.82859. Nevertheless, the same was not observed with TLS and TLS_*v*_, and close values were found among them.

### 3.1. *In Vitro* Biological Assays

The* in vitro* test is important for screening a substance which can be used subsequently in preclinical trials. In addition, it is able to provide initial parameters such as viability and therapeutic targets for subsequent reviews [35].

The materials used in the formulations present well-known safety profiles; however, these mixtures have been shown to form structures which may change the barrier properties of the skin. Therefore, it is important to study the safety of these new systems on the skin.

Erythrocyte membranes have been broadly used for* in vitro* cytotoxicity assays because they are easily isolated by centrifugation and obtained by venipuncture. They represent a good model to evaluate the interaction of drugs with the membrane and thus provide information about changes in the composition of lipids, enzymes, or other membrane proteins [36].

The safety profile was assessed using the hemolysis of red blood cells and* in vitro* cytotoxicity assays. Free RP caused 4.69 ± 0.54% lysis of erythrocyte membranes. TLS and OLS caused lysis of 1.23% ± 0.69 and 1.49 ± 0.35% of the erythrocyte membranes, respectively. The incorporation of RP in LCS—TLS_*v*_ and OLS_*v*_—was 3.19 ± 1.07% and 3.42 ± 0.54%, respectively and showed decreased erythrocyte lysis compared to the free RP. Thus, all systems showed a tolerable hemolysis of erythrocytes. The positive control was represented by 100% hemolysis of erythrocytes using Triton X-100, a known hemolytic agent, thus validating the experiment. The study results indicate that the treatment developed with lipid systems showed less toxicity, which is a potential alternative to therapeutic applications [20].

Similarly, the safety profile of praziquantel (PZQ) was evaluated in studies using red cell hemolysis and showed that the encapsulation of PZQ in nanostructured lipid carriers improved the safety profile of the drug with decreased lysis of erythrocytes in relation to the free PZQ. The systems demonstrated improved efficacy in comparison with free PZQ [37].

**Figure 5:**
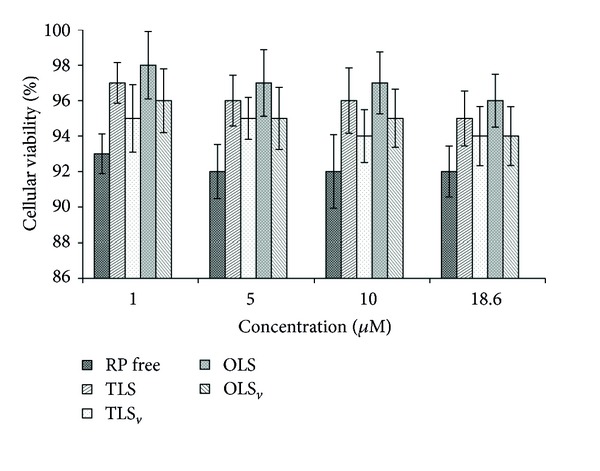
Percentage of cell viability of RP free, transparent liquid system (TLS), opaque liquid system (OLS), RP-loaded transparent liquid system (TLS_*v*_), and RP-loaded opaque liquid system (OLS_*v*_).

*In vitro* cytotoxicity was performed using J-774 mouse macrophages as a cellular model. The data are shown as a percent of cellular viability (Figure 5).

Abbasalipourkabir et al. (2011) say that some factors may influence the cytotoxic effect of the particles: adherence to the cell membrane, particle internalization, and degradation products in the cell culture medium or in the intracellular environment [38]. Nevertheless, cell types may exhibit different changes in susceptibility to particulate carriers since the system contains natural lipids to be well tolerated by the body [39].

Cell viability showed that free RP did not kill normal macrophages presenting a result superior to 92%. Moreover, the RP-unloaded and RP-loaded CLS also did not show toxic activity. Therefore, the results indicate the safety and biocompatibility of the formulations and free RP for eukaryotic cells [37].

**Figure 6:**
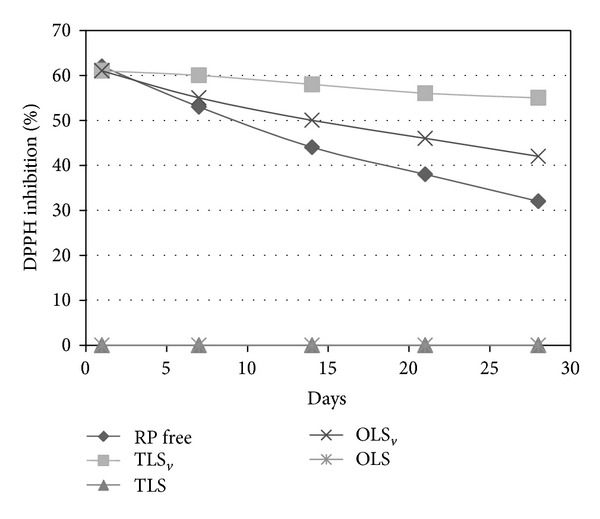
Percentage of inhibition of 2,2-diphenyl-1-picrylhydrazyl (DPPH) radical by formulations over a period of 28 days. RP free: retinyl palmitate free, TLS_*v*_: retinyl palmitate-loaded transparent liquid system, TLS: transparent liquid system, OLS_*v*_: retinyl palmitate-loaded opaque liquid system, and OLS: opaque liquid system.

The formulations with or without the addition of RP were evaluated for* in vitro *antioxidant activity over a period of 28 days. The results obtained are shown in Figure 6.

The antioxidant activity was measured based on the methodology of Blois [21], who uses the stable radical DPPH which is reduced by antioxidants. The results were expressed as DPPH inhibition (%).

It can be seen that over the period of 28 days there was a decrease in antioxidant power for all the experimental groups, with the most marked decrease for the RP free and a statistical difference between the samples (*P* < 0.05). The lesser destabilization of the samples is probably due to increased stabilization of the vitamin in the formulations. Moreover, the antioxidant activity was greater for TLS_*v*_ compared to OLS_*v*_, suggesting increased stability of the vitamin in the lamellar LCS. This effect has been found in the literature; the liquid crystals can protect the formulation while preventing the degradation of the active ingredient [13, 16, 35, 40, 41].

Therefore, all formulations maintained the antioxidant properties of the RP, which makes them promising as vehicles for incorporation of the vitamin, aiming its topical application at the treatment of aging skin.

## 4. Conclusion

The results suggest that the LCS containing polyether functional siloxane (PFS) as surfactant, silicon glycol copolymer (SGC) as oil phase, and water in the ratios 40 : 50 : 10 with RP (TLS_*v*_) presented a lamellar phase and pseudoplastic behavior in rheological analysis.* In vitro* safety profile showed that this formulation is not cytotoxic and* in vitro* antioxidant activity suggested that LCS presented a higher capacity to maintain the antioxidant activity of RP. PFS-based systems may be a promising platform for RP topical application for the treatment of skin aging.

## Figures and Tables

**Table 1:** Index of flow (*η*) and consistency index (*k*) of the formulations.

Formulations	*η*	*k*
OLS	0.28834	19.2766
OLSv	0.82859	5.00762
TLS	0.82742	5.60389
TLSv	0.78206	8.36384

OLS: opaque liquid system, OLSv: RP-loaded opaque liquid system, TLS: transparent liquid systems, and TLSv: retinyl palmitate-loaded transparent liquid systems.
